# Percutaneous treatment of simple renal cysts with 24-h-interval ethanol sclerotherapy

**DOI:** 10.1590/0100-3984.2022.0041

**Published:** 2023

**Authors:** Hakki Muammer Karakas, Gulsah Yildirim

**Affiliations:** 1 Department of Radiology, University of Health Sciences, Fatih Sultan Mehmet Training and Research Hospital, Istanbul, Turkey.; 2 Department of Radiology, University of Health Sciences, Istanbul Training and Research Hospital, Istanbul, Turkey.

**Keywords:** Kidney diseases, cystic, Sclerotherapy/methods, Sclerosing solutions/therapeutic use, Doenças renais císticas, Escleroterapia/métodos, Soluções esclerosantes/uso terapêutico

## Abstract

**Objective:**

To investigate the efficacy of 24-h interval multiple-session ethanol sclerotherapy for the treatment of simple renal cysts.

**Materials and Methods:**

The study sample included 58 patients (mean age, 65.37 ± 11.95 years). We included 76 simple renal cysts that were treated with percutaneous aspiration with a minimum of two sessions of 95% ethanol sclerotherapy in a 24-h interval between sessions. Patients were evaluated at 1, 3, and 6 months after the intervention for the efficacy of the treatment. Treatment success was defined as a complete regression of a cyst or a > 50% reduction in its volume, with no recurrence of symptoms.

**Results:**

The mean preprocedural cyst size was 72.98 ± 25.14 mm, and the mean preprocedural cyst volume was 205.76 ± 244.15 mL. The mean volume of ethanol used in the first sclerotherapy session was 62.76 ± 30.71 mL. The mean fluid accumulation in the cysts at the end of the first 24-h interval was 4.66 ± 7.13 mL. The mean quantity of ethanol used in the second sclerotherapy session was 26.48 ± 22.2 mL. A third sclerotherapy session was required in only 10 (13.2%) of the cysts. The mean follow-up period was 52.84 ± 37.83 months. The rate of complete regression was 97.4% for the whole sample at the end of the follow-up.

**Conclusion:**

Ethanol ablation with 24-h intervals is a safe and effective treatment option in the minimally invasive percutaneous treatment of simple renal cysts.

## Abstract (pt)

**Objetivo:**

Demonstrar a eficácia da escleroterapia com etanol em sessões múltiplas em intervalos de 24 horas no tratamento de cistos renais simples.

**Materiais e Métodos:**

Foram avaliados 76 cistos renais simples de 58 pacientes (média de idade: 65,37 ± 11,95 anos) submetidos a aspiração percutânea e ao menos duas sessões de escleroterapia com etanol 95% num intervalo de 24 horas. Considerou- se sucesso de tratamento a regressão completa ou uma redução > 50% do volume do cisto, sem recorrência dos sintomas. Os pacientes foram reavaliados 1, 3 e 6 meses após o procedimento.

**Resultados:**

Antes do procedimento, a média do tamanho dos cistos foi de 72,98 ± 25,14 mm e a média do volume dos cistos foi de 205,76 ± 244,15 mL. A quantidade média de etanol utilizada na primeira sessão de escleroterapia foi de 62,76 ± 30,71 mL. A média de acúmulo de líquido ao final do intervalo de 24 horas foi de 4,66 ± 7,13 mL, sendo utilizada uma média de quantidade de etanol de 26,48 ± 22,2 mL na segunda sessão de escleroterapia. Uma terceira sessão de escleroterapia foi necessária em apenas 10 (13,2%) dos cistos. Na amostra geral, a taxa de regressão completa foi de 97,4%. O tempo médio de seguimento foi de 52,84 ± 37,83 meses.

**Conclusão:**

A ablação com etanol em intervalo de 24 horas é uma opção de tratamento segura, eficaz e minimamente invasiva no tratamento percutâneo de cistos renais simples.

## INTRODUCTION

Simple renal cysts are common lesions, occurring in 20-50% of the general population, and their incidence increases with age, affecting nearly 40% of people over 70 years of age^(1,2)^. Although their etiology has not been fully elucidated, they are thought to develop secondary to basement membrane changes originating from the convoluted tubule and collecting duct. Most renal cysts are asymptomatic and are diagnosed incidentally. According to the Bosniak classification of renal cysts, simple cysts are classified as category I or II cysts, and the presence of symptoms is the determining factor in the monitoring and treatment of such cysts^(3)^. The decision to treat simple renal cysts depends on whether the cyst is symptomatic, complicated, or both. The main goal is to prevent damage to the adjacent parenchyma due to increased cyst volume^(4)^. Minimally invasive percutaneous treatment of symptomatic renal cysts is a widely used and successful method that can be applied in various ways. Percutaneous aspiration-sclerotherapy is the most widely used treatment option because of their high efficacy, low cost, and the low complication rate^(2)^. Although many different substances are used as sclerosing agents, ethanol in high concentrations (95.0-99.9%) is the preferred agent because of its availability, cost, effectiveness, and ease of use. The level of experience in ultrasound- and computed tomography-guided interventions largely affects the results. However, even then, complete resolution is not achieved in some cases, especially those in which single-session sclerotherapy is used. The reported mean rate of recurrence after single-session sclerotherapy with ethanol is 32 ± 100%^(3,5)^.

The aim of this study was to determine the efficacy of 24-h-interval, multiple-session ethanol sclerotherapy for the treatment of simple renal cysts, regarding the technical and clinical results, as well as the tolerability.

## MATERIALS AND METHODS

This study was approved by the local institutional review board (Reference no. 17073117_050.06.99-80). Written informed consent was obtained from all participating patients. The study was conducted in accordance with the principles of the Declaration of Helsinki.

The inclusion criteria were having a proven simple renal cyst and showing clinical characteristics consistent with renal cysts. Patients with uncorrectable coagulopathy (defined as an international normalized ratio > 1.5 or a platelet count < 50,000/mm^3^) were excluded from the study.

**Figure 1:**
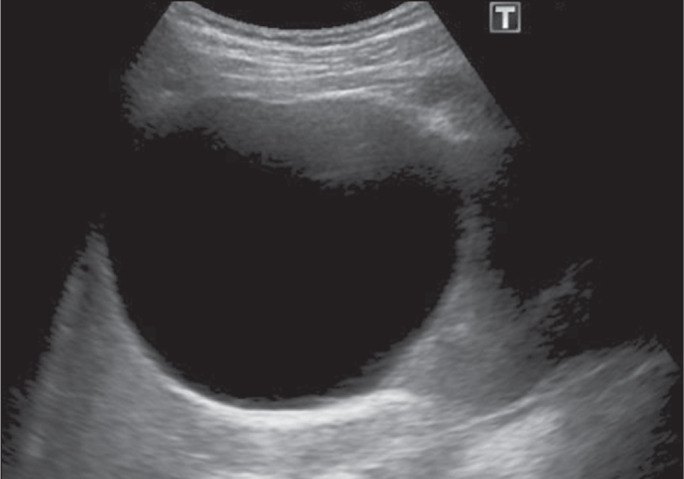
An 80-mm simple renal cyst. This cyst, from which 200 mL of fluid was aspirated, was representative of the average cyst size and volume in our study sample.


All patients were evaluated with ultrasound, and the volumes of their cysts were recorded before the sclerotherapy (Figure 1). The size of each cyst was calculated with the standard ellipsoid formula:

volume=(a×b×c)×0.52

where *a, b*, and *c* are the largest orthogonal diameters.

**Figure 2:**
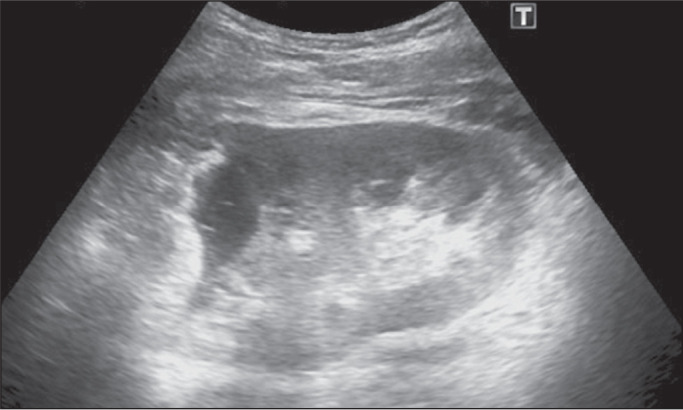
The appearance of the representative cyst during intracavitary injection of 20 mL of the anesthetic agent (prilocaine hydrochloride), a dose that provided effective pain control even in larger cysts.

**Figure 3:**
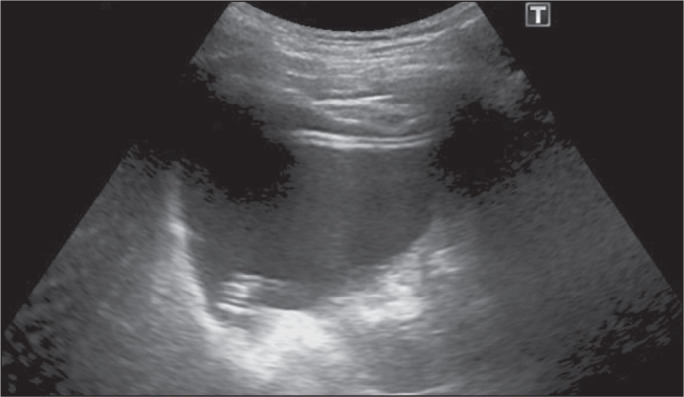
The appearance of the representative cyst and the catheter, after the injection of ethanol at 50% of the aspirated cyst volume.

All patients were monitored before the procedure, regarding vital signs. After the administration of a local anesthetic (2% prilocaine), the cyst was punctured with an 18-G percutaneous entry needle under ultrasound guidance. If there was any suspicion of involvement of the renal collecting system, 300 mg/mL of contrast material, diluted at 1:10 with saline solution, was administered under fluoroscopy. Cystography was performed to identify leakage and communication with the collecting system. In the next step, a 6-8 F locking multi-purpose ethanol-resistant drainage catheter (Skater; Argon Medical Devices, Plano, TX, USA) was placed by using the single-step technique for lesions closer than 4 cm to the skin and the Seldinger technique in the remaining ones. After complete aspiration of the cyst content, intracavitary 5-20 mL of the anesthetic prilocaine hydrochloride (mean, 17.90 ± 4.52 mL) was injected into the cavity, where it was allowed to remain for 4 min (Figure 2). In 81.6% of the cases, 20 mL of the anesthetic was administered. Although the pain response to ethanol sclerotherapy varied among the patients, administration of 20 mL of prilocaine provided effective anesthesia in almost all cases. Following aspiration of the anesthetic agent, 95% ethanol was administered at a volume of 50% of the aspirated cyst volume, not exceeding 100 mL per cyst. The intracavitary ethanol was aspirated, and an equal volume of ethanol was injected immediately thereafter, to prevent the dilution of the ethanol by the residual cyst fluid (Figure 3). Patients were instructed to turn 90° on the table every 5 min to ensure equal contact of the ethanol with all surfaces. The ethanol was aspirated completely after 20 min (i.e. after a full turn), and the catheter was clamped for 24 h. At the end of the 24-h period, the accumulated fluid was measured and the procedure was repeated on the basis of inspection of the volume of the accumulated content. For cases in which the fluid accumulation was ≥ 10 mL, the procedure was repeated with the same amount of ethanol used in the first session; if the accumulation was < 10 mL, ablation was performed with ethanol at 50% of the aspirated cyst volume. The patient rotation step was repeated, and, at the end of the procedure, all of the ethanol was aspirated. If the fluid accumulation was ≥ 10 mL after the second 24-h period, a third ablation session was performed as described above, after which all of the ethanol was aspirated and the catheter was removed. If the amount of fluid accumulated after the second 24-h period was < 10 mL, a third session was not performed but all of the content was aspirated and the catheter was removed.

**Figure 4:**
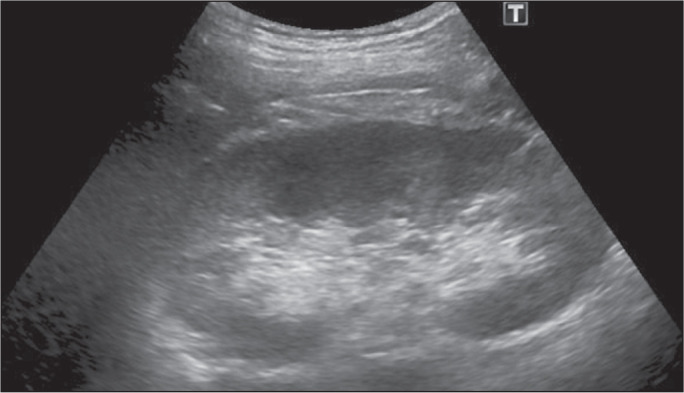
Ultrasound examination at 6 months of follow-up, showing complete regression of the representative cyst.

Patients were initially placed in the prone position. The supine position was avoided, because it would preclude the rotational maneuvers that would allow the anesthetic and sclerosing materials to come into contact with the cyst walls and would require re-entry in each session. Patients were evaluated at 1, 3, and 6 months after the procedure, and the findings at 6 months of follow-up constituted proof of the success or failure of the treatment (Figure 4). Thereafter, the patients were evaluated at 12-month intervals. Treatment success was defined as a ≥ 50% reduction in cyst volume/size with no recurrence of symptoms during follow-up^(6)^.

### Statistical analysis

Statistical evaluation was performed using IBM SPSS Statistics software package, version 25.0 (IBM Corp., Armonk, NY, USA). Data were summarized with the use of descriptive statistics. Continuous variables were reported as mean ± standard deviation and range. The t-test for independent samples was used in order to compare the means between groups. Values of *p* < 0.05 were considered statistically significant. The relationships between variables were evaluated by correlation analysis.

## RESULTS

A total of 76 simple renal cysts, in 58 patients (42 men; 16 women), were treated with percutaneous sclerotherapy between January 2015 and September 2021. The mean age of the patients was 65.4 ±11.9 years (range, 11-82 years. The presenting symptoms were flank/back pain in 53 patients (91.4%) and hypertension in five (8.6%), as shown in Table 1.

**Table 1:** Baseline demographic and clinical characteristics of patients undergoing ethanol sclerotherapy for the treatment of simple renal cysts.

Characteristic	(N = 58)
Age, mean ± SD	65.37 ± 11.95
Female, n (%)	16 (27.6)
Cyst volume (mL), mean ± SD	205.76 ± 244.15
Largest diameter (mm), mean ± SD	72.98 ± 25.14
Presenting symptom, n (%)	
Pain (flank/back)	53 (91.4)
Hypertension	5 (8.6)

SD, standard deviation.

**Figure 5:**
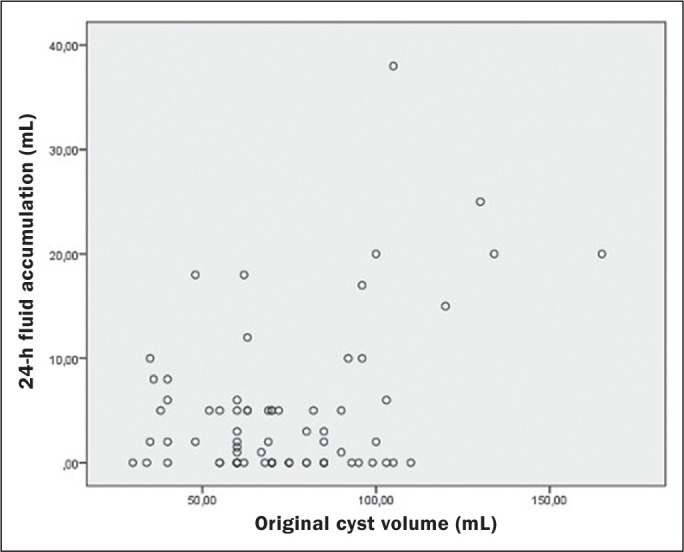
Relationship between the original cyst volume and the 24-h fluid accumulation after ablation.

Prior to the procedure, the mean cyst size was 72.98 ± 25.14 mm (range, 30-165 mm) and the mean cyst volume was 205.76 ± 244.15 mL (range, 20-1,778 mL). Alcohol sclerotherapy with aspiration and injection was performed on all 76 cysts in two or three sessions. In the first sclerotherapy session, the mean volume of ethanol used was 62.76 ± 30.71 mL (range, 8-100 mL). The mean amount of fluid accumulated at the end of the first 24-h period was 4.66 ± 7.13 mL (range, 0-38 mL), and the mean volume of ethanol used in the second sclerotherapy session was 26.48 ± 22.2 mL (4-100 mL). There was no correlation between the initial volume of ethanol administered and 24-h fluid accumulation (Figure 5). A third session was required in only 10 cysts (13.2%). The mean preprocedural cyst volume was 957.66 mL (range, 80-1,778 mL), significantly higher than in the other cysts (*p* < 0.05).

Complete regression was achieved in all but two cysts. One of those two cysts extended to the para-pelvic region, dissecting the kidney. The other cyst was the largest in our sample, in terms of size (165 mm) and volume (1,778 mL). In both of those cases, the cyst diameter was < 30 mm after the treatment. In the sample as a whole, the 6-month regression rate was 97.4%.

From a technical standpoint, only two patients were unable to tolerate the procedure. In both of those cases, the procedure was terminated within the first few minutes of the initial session. In one of those cases, the cyst wall was very thick and it was possible that the anesthetic had not diffused sufficiently to reach the cyst wall. In the other case, ethanol extravasation to the surrounding tissue was minimal.

In all but one case, no pain was observed during or after ethanol exposure. In those cases, no sedation or intravenous analgesics were administered. No major complications were observed during or after the procedure. In one patient, the catheter broke at the surface of the cyst during the withdrawal process after the third session. That patient was started on a long-term course of antibiotic therapy, and no additional complications were observed during follow-up. The mean follow-up time was 52.84 ± 37.83 months (range, 6-120 months). Of the 58 patients evaluated, 41 (70.7%) were followed for more than two years, and there was no recurrence among those patients.

## DISCUSSION

The first-line treatment in simple renal cysts is percutaneous drainage with sclerotherapy, because of its minimally invasive nature. Although the most common symptom requiring intervention is pain (in 80% of cases), approximately 70% of such cysts are asymptomatic^(6)^. Sclerotherapy should be performed as a complement to drainage because cyst fluid inevitably reaccumulates after drainage. The reported success rate of percutaneous cyst drainage alone is 26.4%, and the recurrence rates reported after that treatment range from 30% to 80%^(5,7,8)^. Ethanol effectively kills cells in the cyst membrane through cell membrane lysis, protein denaturation, and vascular occlusion. Fixation, which causes the secretion to stop, theoretically occurs within the first 3 min. The renal parenchyma is preserved because the passage of ethanol through the fibrous capsule takes 4-12 h^(9)^. Complications associated with alcohol injection are related to the entry of ethanol into the bloodstream or accidental intravascular injection. In both cases, varying degrees of intoxication and hypotension may occur. Before the procedure, it should be ensured that the patient has not had recent ethanol exposure and does not have the acetaldehyde dehydrogenase 2 genotype. Exposure to 200 mL of ethanol for 20 min is considered the upper limit, and many studies have used ethanol at a volume of 15-50% of the original cyst volume^(9,10)^. In the present study, we opted to use 95% ethanol at a volume of 50% of the cyst volume aspirated. The mean quantity of 95% ethanol injected in the first sclerotherapy session was 62.76 ± 30.71 mL, and there were no complications. To prevent systemic complications, we limited the total volume of injected to 100 mL.

Two consecutive sessions of sclerotherapy, consisting of 20 min of ethanol exposure per session, with a 24-h interval between the sessions, has a very low complication rate and a high success rate, as well as being a treatment that is well tolerated. The 6-month complete regression rate was found to be 97.4% in the present study, which is similar to rates reported in the literature. Single-session sclerotherapy for the treatment of percutaneous cysts has been reported to achieve complete regression rates ranging from 17% to 94%^(11-14)^. In a comparative study conducted by Chung et al.^(14)^, the success rate reported for single-session sclerotherapy was 57%, compared with 95% for multiple-session sclerotherapy. Hanna et al.^(5)^ followed patients for two years after sclerotherapy and reported a recurrence rate of 32% among those who underwent single-session sclerotherapy, whereas they observed no recurrence among those who underwent two-session, 24-h-interval sclerotherapy. In our study sample, there were also no cases of recurrence during the 2-year follow-up period. Therefore, ethanol sclerotherapy, performed in two sessions with a 24-h interval between sessions, appears to be superior to single-session sclerotherapy.

The main cause of recurrence is insufficient contact between the sclerosing agent and the cyst wall. Therefore, it is recommended that ethanol be applied at a minimum of 30% of the cyst volume and that rotational maneuvers be performed^(13)^. If the cyst is very large, that volume cannot be achieved. In such cases, rotational maneuvers should be performed for 40 min. Based on our previous experience, for sclerotherapy to be effective, it should be repeated even if there is no accumulated fluid after the first session. The volume of ethanol injected should ideally be 50% of that of the accumulated fluid in cases of high-volume accumulation and at least 25% of the original volume for high-volume cysts.

The complication rate of ethanol ablation is extremely low. However, radiologists should be prepared for the possibility of systemic hypotension, increased pulmonary vascular resistance, and myocardial toxicity that may develop due to ethanol-related complications^(15,16)^. All cases should be monitored during the session, especially in terms of blood pressure. In addition, it is important to use an ethanol-resistant catheter to prevent damage to the catheter after ethanol exposure and to avoid possible complications. There have been studies suggesting that ethanol exposure degrades the mechanical integrity of polyurethane catheters^(17)^. However, it has been reported that 70% ethanol solutions do not adversely affect the mechanical properties of polyetherurethane and silicone catheters after exposure for up to 10 weeks^(18)^. In our study sample, there were no complications related to ethanol administration.

The main limitation of our study was the unavailability of a control group, which would have allowed comparison between the efficacy of single-session sclerotherapy and that of percutaneous catheter drainage. However, our professional experience and anecdotal evidence preclude the use single-session sclerotherapy at our center, because it could result in incomplete treatment.

In conclusion, imaging-guided ethanol ablation performed in consecutive sessions with 24-h intervals between sessions appears to be a highly successful, safe procedure. It also has very good tolerability. It is recommended as the first step in the treatment of symptomatic simple renal cysts.
